# Causal Insights Into Crohn's Disease: The Role of Oxidative Stress and Genetic Variants

**DOI:** 10.1111/jcmm.71257

**Published:** 2026-07-15

**Authors:** Juan Yang, Lida Zhang, Xiaqing Wang, Yuxiu Yang

**Affiliations:** ^1^ Gastroenterology Department FuWai Central China Cardiovascular Hospital Zhengzhou Henan Province China; ^2^ Gastroenterology Department Henan Provincial People's Hospital Zhengzhou Henan Province China

**Keywords:** CD, differential expression, mendelian randomization, oxidative stress, protein–protein interaction networks

## Abstract

Crohn's disease (CD) is a chronic inflammatory condition of the gastrointestinal tract, where oxidative stress is a significant contributing factor to its pathogenesis. This study utilized multi‐omics data, including RNA sequencing from the GSE216447 dataset and three genome‐wide association studies (GWAS) datasets (ieu‐a‐10, ieu‐a‐11, ieu‐a‐13), to investigate the molecular networks related to oxidative stress in CD. Differential expression analysis was performed using DESeq2, followed by pathway enrichment analysis with clusterProfiler. Protein–protein interaction (PPI) networks were constructed using the STRING database. Mendelian Randomization (MR) analysis was conducted using TwoSampleMR and MRMix to identify causal relationships between genetic variants and CD. Quantitative real‐time polymerase chain reaction (qPCR) was further applied to verify key differentially expressed genes (DEGs), including FASN, HMGCR, ASCC3, CD101, ELOVL6, PHLDA2, PHLDA3, and SCPEP1, in intestinal mucosal samples from both inactive and active CD patients. The analysis identified 64 up‐regulated and 46 down‐regulated differentially expressed genes (DEGs) in response to H_2_O_2_ intervention. Key pathways related to oxidative stress, including the p53 signalling pathway and steroid biosynthesis, were significantly enriched. Consistent with transcriptomic data, qPCR confirmed that FASN and HMGCR were significantly upregulated in inactive CD, while ASCC3, CD101, ELOVL6, PHLDA2, PHLDA3, and SCPEP1 were markedly increased in active CD (all *p* < 0.05). The MR analysis revealed that in the dataset ieu‐a‐10, ABCB9 and OSGIN1 were identified as having a significant causal relationship with CD using TwoSampleMR, while only OSGIN1 was significant in MRMix. In dataset ieu‐a‐11, ARL4C, CD101, HMGCR, and IL24 were found to be significantly associated with CD, with overlapping findings between TwoSampleMR and MRMix. For dataset ieu‐a‐13, ACTA2 and CD101 were consistently identified as significant, suggesting their potential roles in CD pathogenesis. The findings highlight the crucial involvement of oxidative stress‐related molecular networks in CD and underscore the utility of Mendelian Randomization in elucidating causal genetic factors. qPCR validation confirmed persistent upregulation of lipid metabolism genes in inactive CD and significant elevation of inflammation‐related genes in active CD, reinforcing the link between oxidative stress and disease activity.

## 1 Introduction

CD is a chronic inflammatory bowel disease characterized by periods of relapse and remission, resulting in significant morbidity. The pathogenesis of CD is complex, involving a multifaceted interplay between genetic predispositions, immune responses, environmental factors, and oxidative stress. Recent studies have highlighted the central role of oxidative stress in the development and progression of CD. Oxidative stress, defined as the imbalance between the production of reactive oxygen species (ROS) and the body's ability to neutralize them, is implicated in disrupting intestinal barrier function, promoting inflammatory responses, and altering gut microbiota—key elements in the pathogenesis of CD [1].

The significance of oxidative stress in CD is further underscored by the presence of elevated oxidative markers in CD patients, even during clinical remission. This persistent oxidative stress suggests a sustained state of inflammation at the molecular level, contributing to the chronic nature of the disease and its associated complications [2]. Moreover, oxidative stress interacts with various molecular pathways, including those related to immune regulation, metabolism, and epigenetic modifications, exacerbating the inflammatory processes in CD [3, 4].

Given the critical role of oxidative stress in CD, understanding the molecular networks involved is essential for identifying potential therapeutic targets. Multi‐omics approaches, integrating data from genomics, transcriptomics, proteomics, and metabolomics, provide a comprehensive view of these molecular networks. These approaches can reveal the intricate interactions between genes, proteins, and metabolites that drive the disease process. The application of Mendelian Randomization (MR) analysis in this context offers a powerful tool to infer causal relationships between genetic variants and disease outcomes. By leveraging genetic data, MR can help identify specific molecular pathways that contribute to CD, providing insights into the mechanisms through which oxidative stress influences the disease [5].

This study aims to explore the molecular networks underlying oxidative stress in CD using a multi‐omics approach. By integrating transcriptomic data with MR analysis, we seek to identify key genes and pathways involved in the oxidative stress response in CD. Understanding these pathways could lead to the development of targeted therapies that modulate oxidative stress, potentially mitigating the inflammatory burden in CD patients.

## 2 Methods

### 2.1 Data Sources

The dataset GSE216447 involves RNA sequencing of primary human epidermal melanocytes treated with H_2_O_2_ to investigate the signalling mechanisms induced by oxidative stress. The study identified the upregulation of cell death and type 1 interferon‐related genes, providing insights into H_2_O_2_‐induced melanocyte signalling [6]. The analysis incorporated data from three distinct GWAS datasets focused on CD across different populations. The ieu‐a‐10 dataset, sourced from a European population study by Jostins et al., 2012, included 14,763 cases and 15,977 controls [7]. The ieu‐a‐11 dataset, from a study by Liu et al., 2015, focused on an East Asian population with 1690 cases and 3719 controls [8]. Lastly, the ieu‐a‐13 dataset, also by Liu et al., 2015, targeted a South Asian population with 184 cases and 990 controls. All datasets were aligned to the HG19/GRCh37 human genome build, facilitating cross‐population comparisons.

### 2.2 Differential Expression Analysis

Differential gene expression analysis was conducted using the DESeq2 package, which facilitates the moderated estimation of fold changes and dispersion for RNA‐seq data. DESeq2 employs shrinkage estimators for dispersion and fold changes, improving the stability and interpretability of the results, particularly in datasets with small sample sizes or low read counts [PMID:25516281].

### 2.3 Gene Ontology and KEGG Pathway Enrichment Analysis

The identification and enrichment analysis of GO biological processes (BP), cellular components (CC), and molecular functions (MF) was performed using the clusterProfiler package. This tool enables statistical analysis and visualization of functional profiles of gene clusters, aiding in the interpretation of the biological significance of DEGs. The KEGG pathway enrichment analysis was also carried out using clusterProfiler, allowing for comprehensive functional annotation of the identified pathways [9, 10].

### 2.4 Gene Set Variation Analysis

Gene Set Variation Analysis (GSVA) was employed to assess variations in pathway activity across the sample population in an unsupervised manner. This method was particularly useful in evaluating the enrichment of predefined gene sets related to specific biological pathways under different conditions [11].

### 2.5 Protein–Protein Interaction Network Construction

The PPI network was constructed using the STRING database, a comprehensive resource for exploring protein association networks. STRING integrates both direct (physical) and indirect (functional) protein associations, enabling detailed analysis of the interaction networks that DEGs may participate in by Szklarczyk et al., 2023 [12].

### 2.6 Study Design and MR Assumptions

This study utilized MR to explore the causal relationships between genetic variants and CD using both TwoSampleMR [13] and MRMix [14] methodologies. MR leverages genetic variants as instrumental variables (IVs) to infer causality between an exposure (in this case, gene expression or genetic variation) and an outcome (CD), under three critical assumptions: (1) the IVs are robustly associated with the exposure, (2) the IVs are not associated with any confounders of the exposure‐outcome relationship, and (3) the IVs affect the outcome only through the exposure of interest. These assumptions were rigorously tested to ensure the validity of the causal inferences.

### 2.7 Selection of Instrumental Variables

Instrumental variables were selected from the GWAS datasets using stringent criteria to ensure they were strongly associated with CD (*p* < 5 × 10^−8^) and were independent (not in linkage disequilibrium). The gwasrapidd R package [15] was employed to interface with the GWAS Catalogue, following best practices for IV selection and eQTL integration, as outlined by Võsa et al., 2021 [16], and Zhu et al., 2016 [17].

### 2.8 Quantitative Real‐Time PCR (qPCR) Validation

To validate the RNA‐seq findings, qPCR was performed on intestinal mucosal samples from 20 healthy controls, 22 inactive CD patients, and 24 active CD patients. Total RNA was extracted using TRIzol reagent, and cDNA was synthesized via reverse transcription. qPCR was conducted using SYBR Green Master Mix on a real‐time PCR system. The relative mRNA expression levels of FASN, HMGCR, ASCC3, CD101, ELOVL6, PHLDA2, PHLDA3, and SCPEP1 were normalized to GAPDH. The 2^(‐ΔΔCt) method was used for quantification, and differences between groups were analysed using an unpaired *t*‐test. *p* < 0.05 was considered statistically significant.

### 2.9 Statistical Analyses

The MR analysis was conducted using the TwoSampleMR package, complemented by the MRMix approach to assess robustness across different assumptions. The primary analysis method was Inverse Variance Weighted (IVW), known for its precision, while MR Egger was applied to detect horizontal pleiotropy. Funnel and scatter plots were generated to visualize the relationships and potential biases. The *p*‐values, confidence intervals (CI), and odds ratios (OR) were calculated to quantify the causal effects, providing comprehensive insights into the genetic determinants of CD.

## 3 Results

### 3.1 DEGs Analysis of H_2_O_2_ Intervention

**FIGURE 1:**
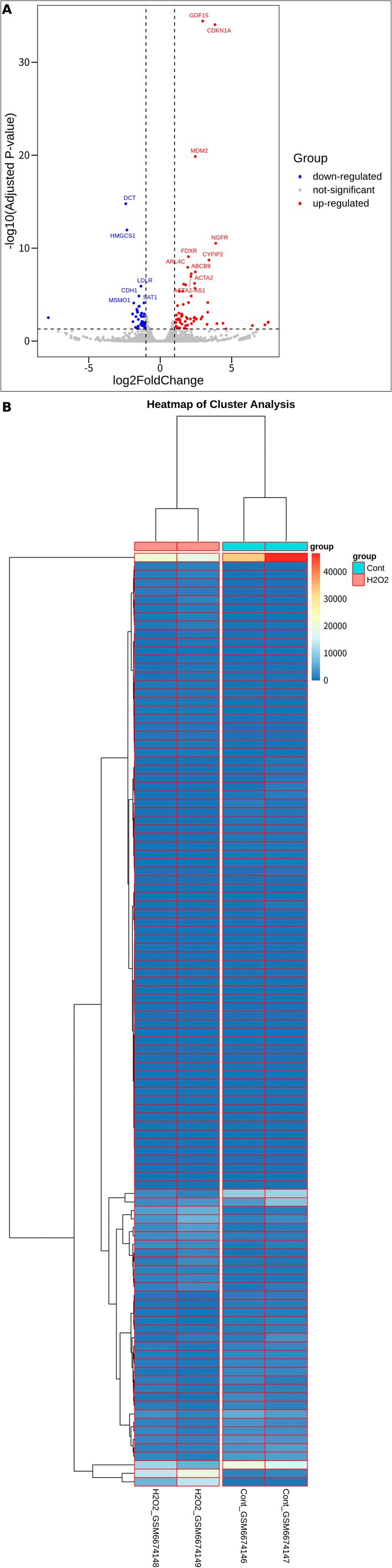
DEGs Analysis. (A) Volcano Plot of DEGs After H_2_O_2_ Intervention. (B) Heatmap Clustering of DEGs Based on H_2_O_2_ Intervention.

The total number of up‐regulated DEGs obtained from DEseq2 analysis is 64, indicating a significant change in gene expression under the studied conditions. Among these up‐regulated genes, the top 10 with the highest log fold changes are GDF15 with a logFC of 2.97 and an adjusted *p*‐value of 3.68e‐35, followed by CDKN1A with a logFC of 3.83 and an adjusted *p*‐value of 8.92e‐35, MDM2 with a logFC of 2.46 and an adjusted *p*‐value of 1.38e‐20, NGFR with a logFC of 3.88 and an adjusted *p*‐value of 2.97e‐11, FDXR with a logFC of 1.97 and an adjusted *p*‐value of 8.15e‐10, CYFIP2 with a logFC of 3.41 and an adjusted *p*‐value of 1.91e‐09, ARL4C with a logFC of 1.93 and an adjusted *p*‐value of 1.14e‐08, ABCB9 with a logFC of 2.46 and an adjusted *p*‐value of 3.58e‐08, ACTA2 with a logFC of 2.17 and an adjusted *p*‐value of 5.72e‐08, and finally ACTA2‐AS1 with a logFC of 2.14 and an adjusted *p*‐value of 1.11e‐07. The total number of down‐regulated DEGs obtained from DEseq2 is 46, indicating that a significant proportion of the differentially expressed genes analysed showed decreased expression levels in the studied samples. Specifically, among the top 10 down‐regulated DEGs identified by DEseq2, DCT (logFC: −2.41, adjusted P Value: 1.63e‐15) was the most significantly down‐regulated, followed closely by HMGCS1 (logFC: −2.33, adjusted P Value: 1.10e‐12), LDLR (logFC: −1.35, adjusted P Value: 1.23e‐06), CDH1 (logFC: −1.49, adjusted P Value: 1.41e‐05), SAT1 (logFC: −1.14, adjusted P Value: 7.65e‐05), MSMO1 (logFC: −1.85, adjusted P Value: 7.92e‐05), ELOVL6 (logFC: −1.47, adjusted P Value: 0.00018), FXYD3 (logFC: −1.49, adjusted P Value: 0.00019), KMO (logFC: −1.64, adjusted P Value: 0.00038), and OPN3 (logFC: −1.61, adjusted P Value: 0.00056) (Figure 1A and Table 1). Furthermore, clustering analysis based on DEGs was conducted, successfully distinguishing between the intervention group and the control group (Figure 1B).

**TABLE 1:** Top 10 up‐regulated and down‐regulated degs in response to H_2_O_2_ intervention.

Symbol	Basemean	Log2foldchange	Lfcse	Stat	*p*	Padj
ABCB9	383.18861617099	2.45570715306996	0.368951532731863	6.65590717264929	2.81557925074416e‐11	3.57944590147105e‐08
ACTA2	393.747673412912	2.16950702754619	0.330088104412875	6.57250897121869	4.94744056039215e‐11	5.71789198584231e‐08
ACTA2‐AS1	387.374068058519	2.14063326721248	0.331366683915753	6.46001354727839	1.04693608372921e‐10	1.10914153603745e‐07
ARL4C	2110.86126984379	1.92633259180417	0.281725450154906	6.8376236181182	8.05176022998771e‐12	1.13735586448704e‐08
CDH1	3082.6070691315	−1.48709930639037	0.266209934708632	−5.5861901172772	2.32105137398912e‐08	1.40512029131065e‐05
CDKN1A	4967.48360219884	3.83286015465099	0.295074415396018	12.9894696207631	1.40396624172732e‐38	8.92431141553971e‐35
CYFIP2	172.264245329092	3.4088946420384	0.479782987123767	7.10507611467062	1.20256245398093e‐12	1.91102205968244e‐09
DCT	6352.25450826769	−2.40864990457844	0.270348483347845	−8.90942636241589	5.12972803029482e‐19	1.63035581122845e‐15
ELOVL6	471.953677928546	−1.46573774058812	0.28877945067727	−5.07563033709823	3.86213506989634e‐07	0.000175354725512829
FDXR	619.709792612352	1.97383029119025	0.272631088271479	7.23993108674665	4.48912782963174e‐13	8.1528974425869e‐10
FXYD3	1022.39550966304	−1.49118960401628	0.295025493828463	−5.05444320985801	4.31648170329459e‐07	0.000189225627220635
GDF15	8446.11148382589	2.97248763057243	0.226737983973438	13.1097912157526	2.89393995347058e‐39	3.67906586284715e‐35
HMGCS1	1304.1568962375	−2.32881922602638	0.286495780726397	−8.12863358797733	4.34156485057301e‐16	1.10388627890669e‐12
KMO	284.33277299595	−1.63785484989857	0.333475210415537	−4.91147407286339	9.03942415945889e‐07	0.000383060664464003
LDLR	2957.17405665396	−1.35023700166241	0.223555173114334	−6.03983787470598	1.54269153813051e‐09	1.22576484526582e‐06
MDM2	2945.83922739246	2.45661270781579	0.24198337265726	10.1519897042483	3.2467538652511e‐24	1.37586606296457e‐20
MSMO1	1362.21105930672	−1.85148865025497	0.352958262931623	−5.24563055947965	1.55748719504377e‐07	7.9201338842366e‐05
NGFR	149.800313511657	3.8849864013947	0.504798271178284	7.69611669296427	1.40263723761328e‐14	2.9719545336296e‐11
OPN3	288.310458642503	−1.60531143001117	0.332322906553969	−4.83057712348958	1.36137868325494e‐06	0.000558297006458713
SAT1	29774.7441620342	−1.14107860099588	0.216954193658646	−5.25953696378528	1.44418597560152e‐07	7.64997346159254e‐05

Abbreviation: Log2 Fold Change: The logarithm (base 2) of the fold change in gene expression between two conditions. *p*‐value: The probability value indicating the significance of the observed difference. Adjusted *p*‐value: The *p*‐value after adjusting for multiple testing, also known as the false discovery rate (FDR).

### 3.2 GO and KEGG Pathway Analysis

**FIGURE 2:**
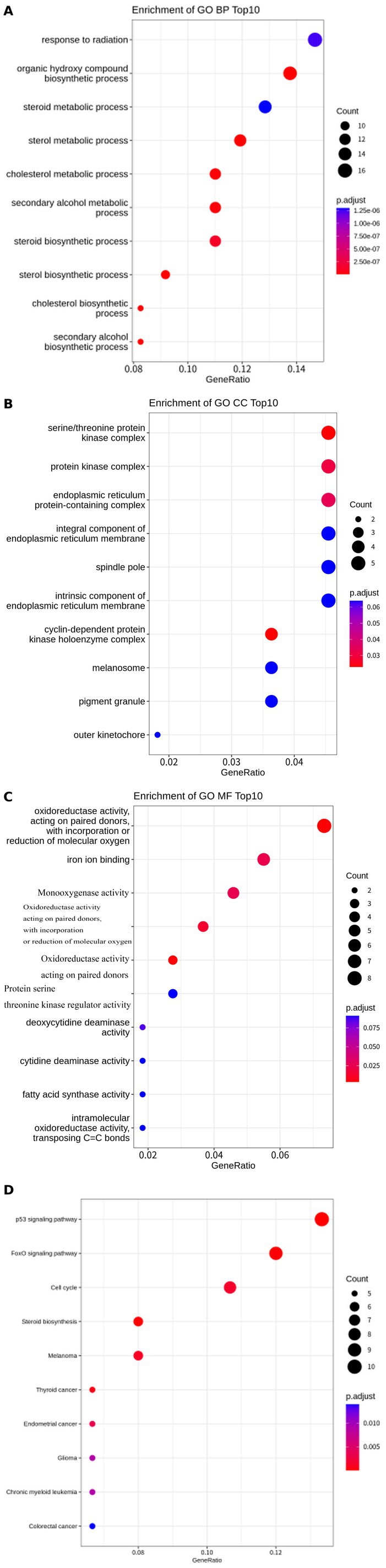
Pathway Analysis with Dotplots. (A) Biological Process (BP) Pathways. (B) Cellular Component (CC) Pathways. (C) Molecular Function (MF) Pathways. (D) KEGG Pathways.

**FIGURE 3:**
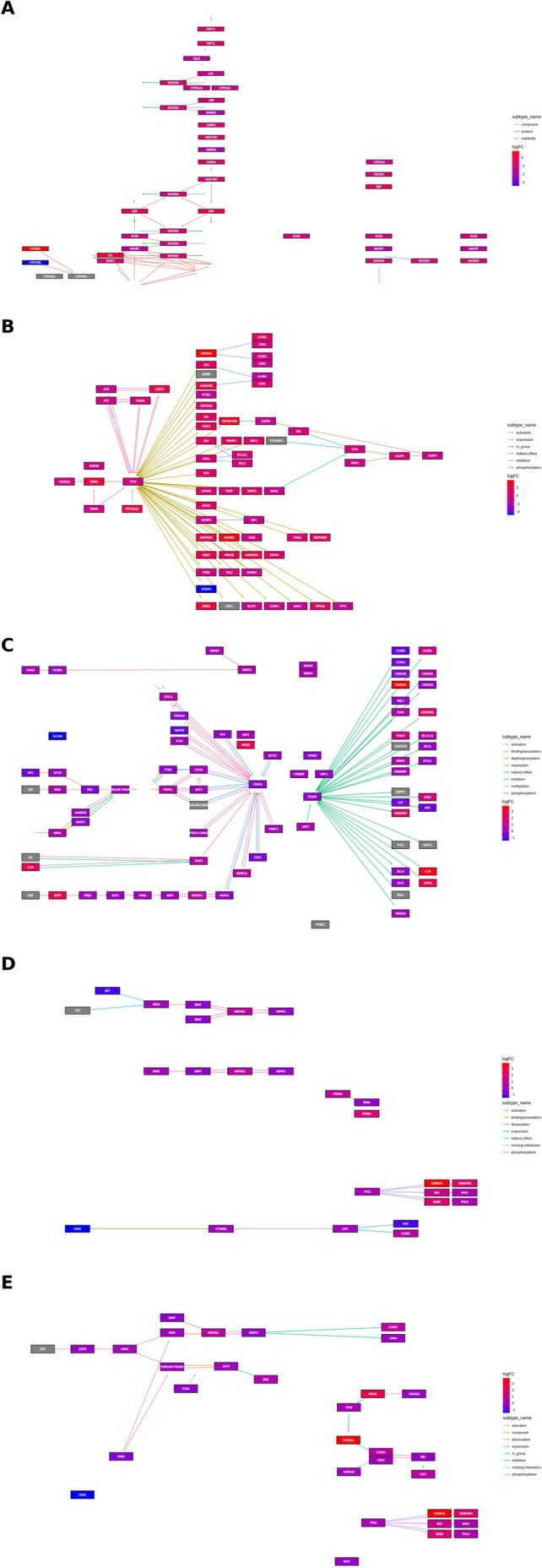
Visualization of Top 5 KEGG Pathways. (A) p53 Signalling Pathway (hsa04115). (B) Steroid Biosynthesis (hsa00100). (C) FoxO Signalling Pathway (hsa04068). (D) Thyroid Cancer Pathway (hsa05216). (E) Melanoma Pathway (hsa05218).

A total of 109 GO‐BP pathways were identified with a threshold of p.adjust < 0.05 by DEseq2 and clusterProfiler. The top 10 GO‐BP pathways include the sterol biosynthetic process (GO:0016126) with 10/109 genes, the sterol metabolic process (GO:0016125) with 13/109 genes, and the organic hydroxy compound biosynthetic process (GO:1901617) with 15/109 genes, among others (Figure 2A). Four GO‐CC pathways were identified with a threshold of p.adjust < 0.05 by DEseq2. The top pathways include the cyclin‐dependent protein kinase holoenzyme complex (GO:0000307), serine/threonine protein kinase complex (GO:1902554), protein kinase complex (GO:1902911), and the endoplasmic reticulum protein‐containing complex (GO:0140534), indicating the importance of these complexes in cellular processes (Figure 2B). In the GO‐MF analysis, five pathways were identified with a threshold of p.adjust < 0.05. Notable pathways include oxidoreductase activity, acting on paired donors, with incorporation or reduction of molecular oxygen (GO:0016705) and iron ion binding (GO:0005506) (Figure 2C). For KEGG pathways, 22 were identified with a threshold of p.adjust < 0.05. The top pathways include the p53 signalling pathway, steroid biosynthesis, and the FoxO signalling pathway, with significant gene ratios and adjusted *p*‐values (Figure 2D), and the top 5 KEGG pathways (hsa04115, hsa00100, hsa04068, hsa05216, hsa05218) were visualized using ggkegg (Figure 3A–E).

### 3.3 GSVA Analysis of H_2_O_2_ Intervention

**FIGURE 4:**
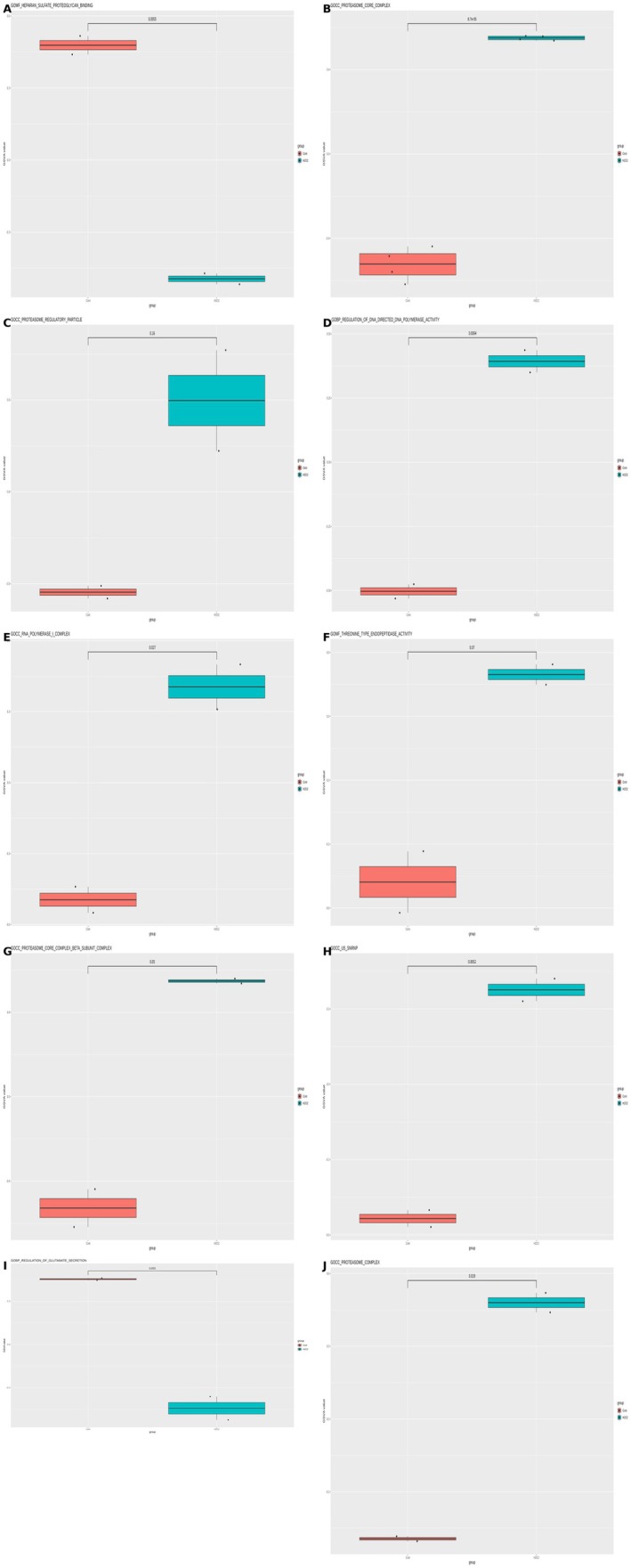
GO Pathway Analysis Using GSVA. (A) Regulation of DNA‐Directed DNA Polymerase Activity (GO‐BP). (B) Proteasome Regulatory Particle (GO‐CC). (C) Proteasome Core Complex (GO‐CC). (D) Threonine‐Type Endopeptidase Activity (GO‐MF). (E) Proteasome Complex (GO‐CC). (F) Regulation of Glutamate Secretion (GO‐BP). (G) U5 snRNP Complex (GO‐CC). (H) Proteasome Core Complex Beta Subunit Complex (GO‐CC). (I) Heparan Sulfate Proteoglycan Binding (GO‐MF). (J) RNA Polymerase I Complex (GO‐CC).

GSVA analysis revealed significant results across 11 GO pathways, such as Regulation of Dna Directed Dna Polymerase Activity, Proteasome Regulatory Particle, Proteasome Core Complex, and Myeloid Dendritic Cell Differentiation had a score of 0.52 and an associated value of −2.97, suggesting a potential involvement in this biological process. Additionally, the cellular response to increased oxygen levels pathway, while having a lower score (0.53), showed higher significance (−2.98), indicating its relevance in the biological context. Pathways related to platelet dense granule and proton transporting two sector atpase complex catalytic domain showed negative scores, suggesting potential downregulation in these processes compared to the upregulated pathways (Figure 4A–4J) (Table 2).

**TABLE 2:** GO pathways related to H_2_O_2_ intervention based on GSVA analysis of degs.

Pathway	Logfc	Aveexpr	T	*p*	Adj.P.Val	B
GO‐CC_PROTEASOME_CORE_COMPLEX	1.072233832943	0.0145184741181867	5.79384278709694	0.000158445466604496	0.455690077361433	0.190625026978281
GO‐CC_PROTEASOME_CORE_COMPLEX_BETA_SUBUNIT_COMPLEX	1.07642094403109	0.0102208269942418	5.7077139395699	0.0001786566270574	0.455690077361433	0.121949821990714
GO‐CC_PROTEASOME_COMPLEX	0.973766873971313	−0.00677267120593099	5.25764652935348	0.000340275603499158	0.455690077361433	−0.258145348962264
GO‐MF_HEPARAN_SULFATE_PROTEOGLYCAN_BINDING	−0.972268157664902	−0.00795856099731226	−5.24521747188167	0.000346525483109323	0.455690077361433	−0.269160518803141
GO‐MF_THREONINE_TYPE_ENDOPEPTIDASE_ACTIVITY	0.974031229177808	0.0093038664228795	4.96537919023817	0.000525099792742691	0.455690077361433	−0.524750522976158
GO‐CC_U5_SNRNP	0.910576155617972	−0.0795155072406725	4.89658162769885	0.000582592485610557	0.455690077361433	−0.58983439713688
GO‐BP_REGULATION_OF_DNA_DIRECTED_DNA_POLYMERASE_ACTIVITY	0.896439656952549	−0.0548851262689698	4.82734701379557	0.00064724896843963	0.455690077361433	−0.656236015404684
GO‐BP_REGULATION_OF_GLUTAMATE_SECRETION	−0.894827006229005	0.00516798678264049	−4.76574110293973	0.000711199521316964	0.455690077361433	−0.71608631567551
GO‐CC_RNA_POLYMERASE_I_COMPLEX	0.899993056061441	−0.0451456040222493	4.72236654768086	0.000760225845256188	0.455690077361433	−0.758658041850577
GO‐CC_PROTEASOME_REGULATORY_PARTICLE	1.04229281683884	−0.0245328144888825	4.71606627625681	0.000767639870702241	0.455690077361433	−0.764871508643262

Abbreviation: t‐Statistic: The test statistic value from the pathway analysis. *p*‐value: The probability value indicating the significance of the pathway enrichment. Adjusted *p*‐value: The *p*‐value after adjusting for multiple testing, indicating the false discovery rate (FDR). B‐Statistic: The log‐odds that a particular pathway is differentially enriched.

### 3.4 PPI Network Analysis

**FIGURE 5:**
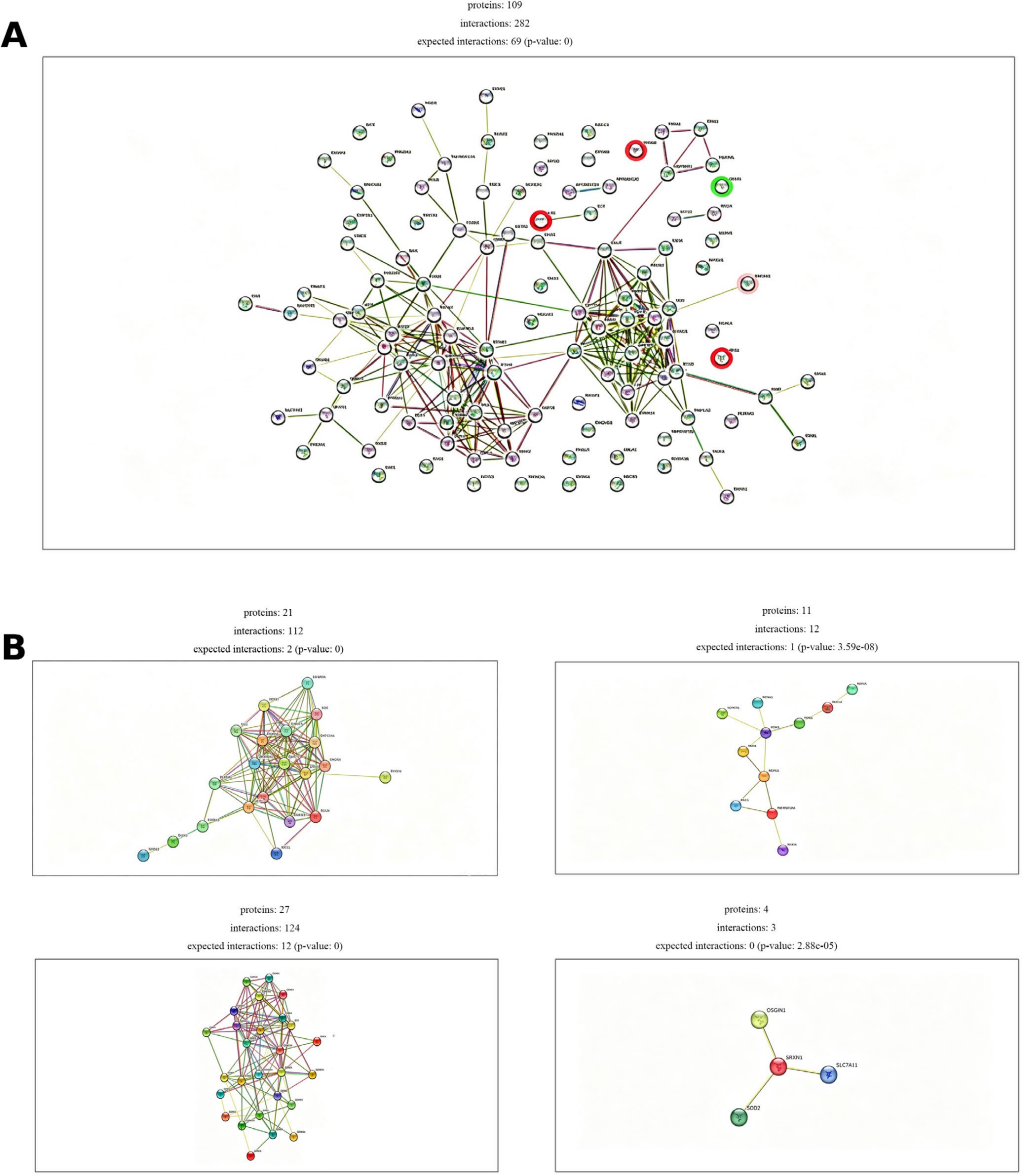
Protein–Protein Interaction Network Analysis of the DEGs of H_2_O_2_ intervention. (A) PPI Network of All DEGs. (B) PPI Network Divided into Four Interconnected Clusters.

In the DEG analysis following H_2_O_2_ intervention, we not only identified significant changes in gene expression but also further explored the potential interactions among these DEGs using PPI network analysis. PPI networks are crucial for understanding the complex signalling pathways and biological processes within cells, providing insights into gene functions and their interactions under specific conditions. Using the R package String_db, a PPI network was constructed (Figure 5A), categorizing DEGs into four interconnected clusters (Figure 5B). This network helps us to delve deeper into the molecular mechanisms occurring within cells after H_2_O_2_ treatment and how different genes work together.

### 3.5 Differential Expression Analysis on of H_2_O_2_ Intervention DEGs in CD Patients

**FIGURE 6:**
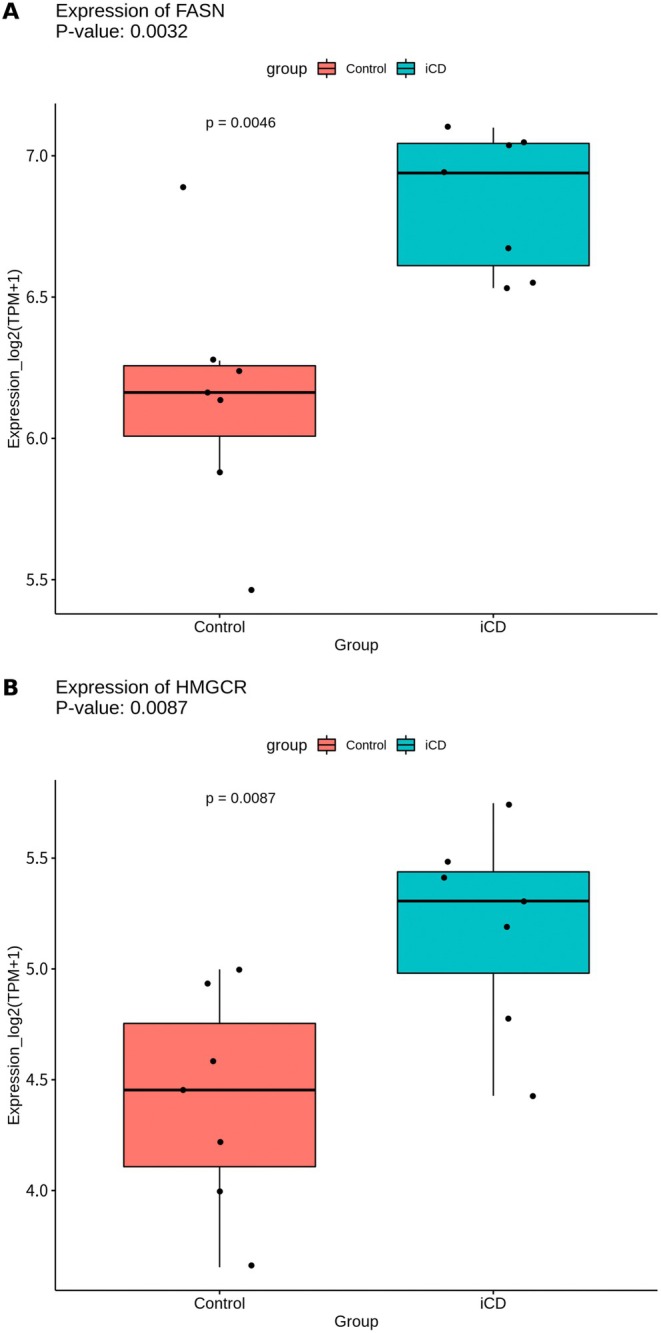
Differential Expression of Top 100 DEGs in Inactive CD Transcriptome. (A) FASN Expression Boxplot. (B) HMGCR Expression Boxplot.

**FIGURE 7:**
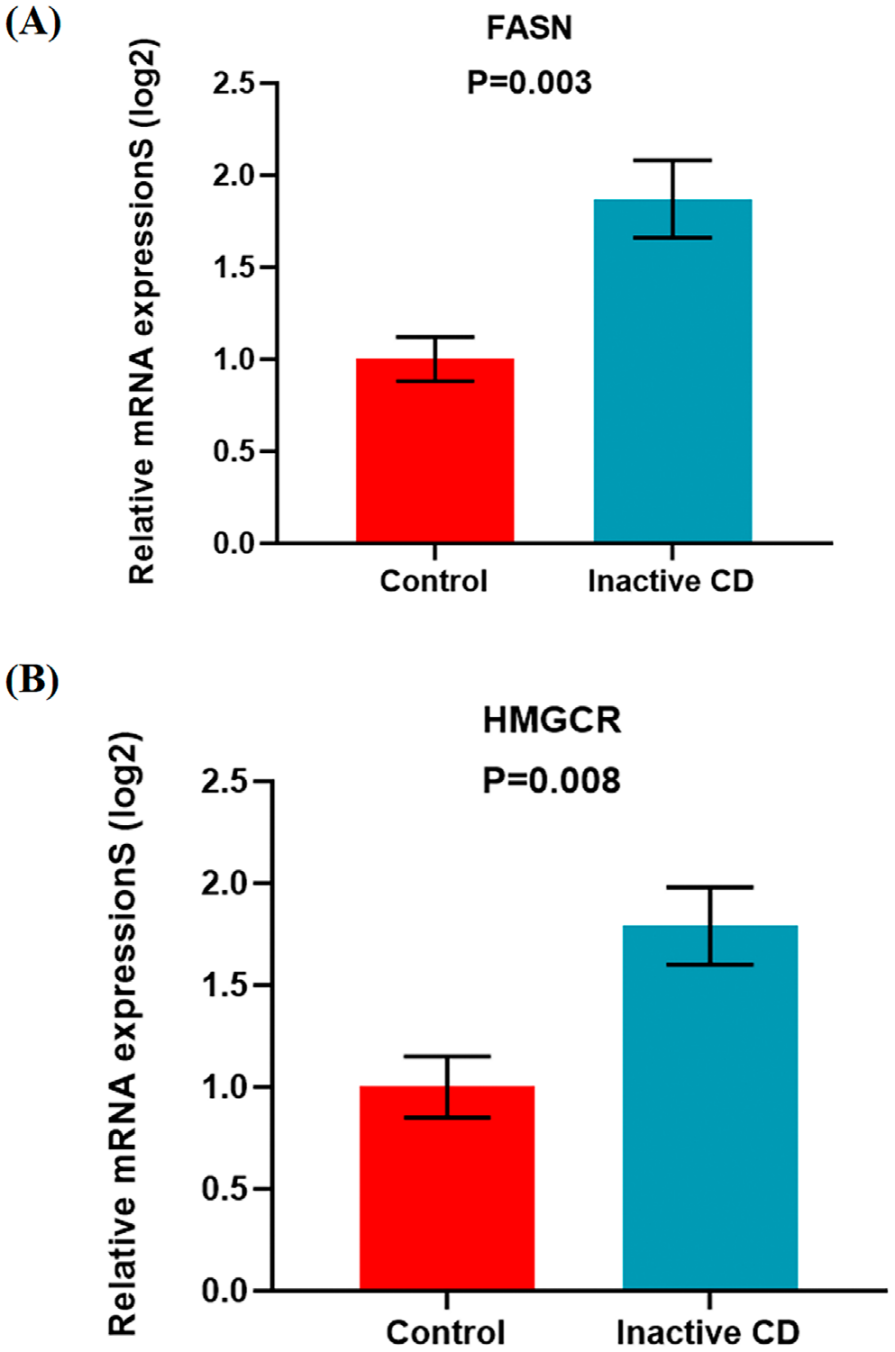
Validation of FASN and HMGCR expression in inactive CD by qPCR. (A) FASN mRNA expression; (B) HMGCR mRNA expression.

Examining the top 100 DEGs in the CD RNA‐seq dataset GSE217163 for inactive samples, we found that 20 genes show differential expression levels in CD, with *p*‐values ranging from 0.0032 for FASN to 0.0498 for PRELP. Notably, the differential expression levels of these genes varied significantly across individuals with CD compared to healthy controls, suggesting that these genes may play crucial roles in the disease's pathogenesis and potentially serve as biomarkers or therapeutic targets. Among them, FASN and HMGCR exhibited the most significant upregulation in inactive CD (ICD) compared with healthy controls (Figure 6). Specifically, FASN expression was markedly increased in ICD patients (*p* = 0.0032; Figure 6A), and HMGCR mRNA levels were also significantly elevated in the inactive CD group (*p* = 0.0087; Figure 6B). To confirm the RNA‐seq findings, qPCR was performed on intestinal mucosal samples from 20 healthy controls and 22 patients with inactive CD. Consistent with transcriptomic data, FASN and HMGCR exhibited the most significant upregulation in inactive CD (ICD) compared with healthy controls (Figure 6). Specifically, FASN expression was markedly increased in ICD patients (*p* = 0.003; Figure 7A), and HMGCR mRNA levels were also significantly elevated in the inactive CD group (*p* = 0.0087; Figure 7B). For example, FASN was found to have a *p*‐value of 0.0032, indicating a significant downregulation in CD samples, while PRELP showed a relatively lower degree of differential expression with a *p*‐value of 0.0498 (Figure 10A,B).

**FIGURE 8:**
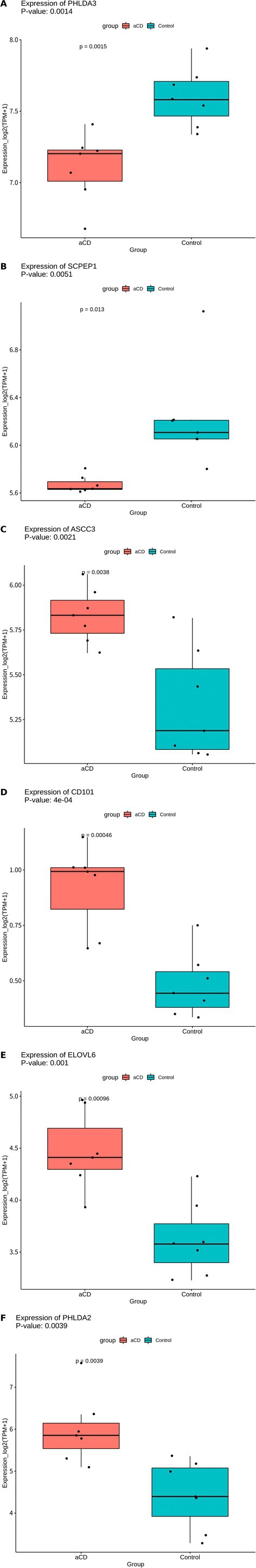
Differential Expression of Top 100 DEGs in Active CD Transcriptome. (A) ASCC3 Expression Boxplot. (B) CD101 Expression Boxplot. (C) ELOVL6 Expression Boxplot. (D) PHLDA2 Expression Boxplot. (E) PHLDA3 Expression Boxplot. (F) SCPEP1 Expression Boxplot.

**FIGURE 9:**
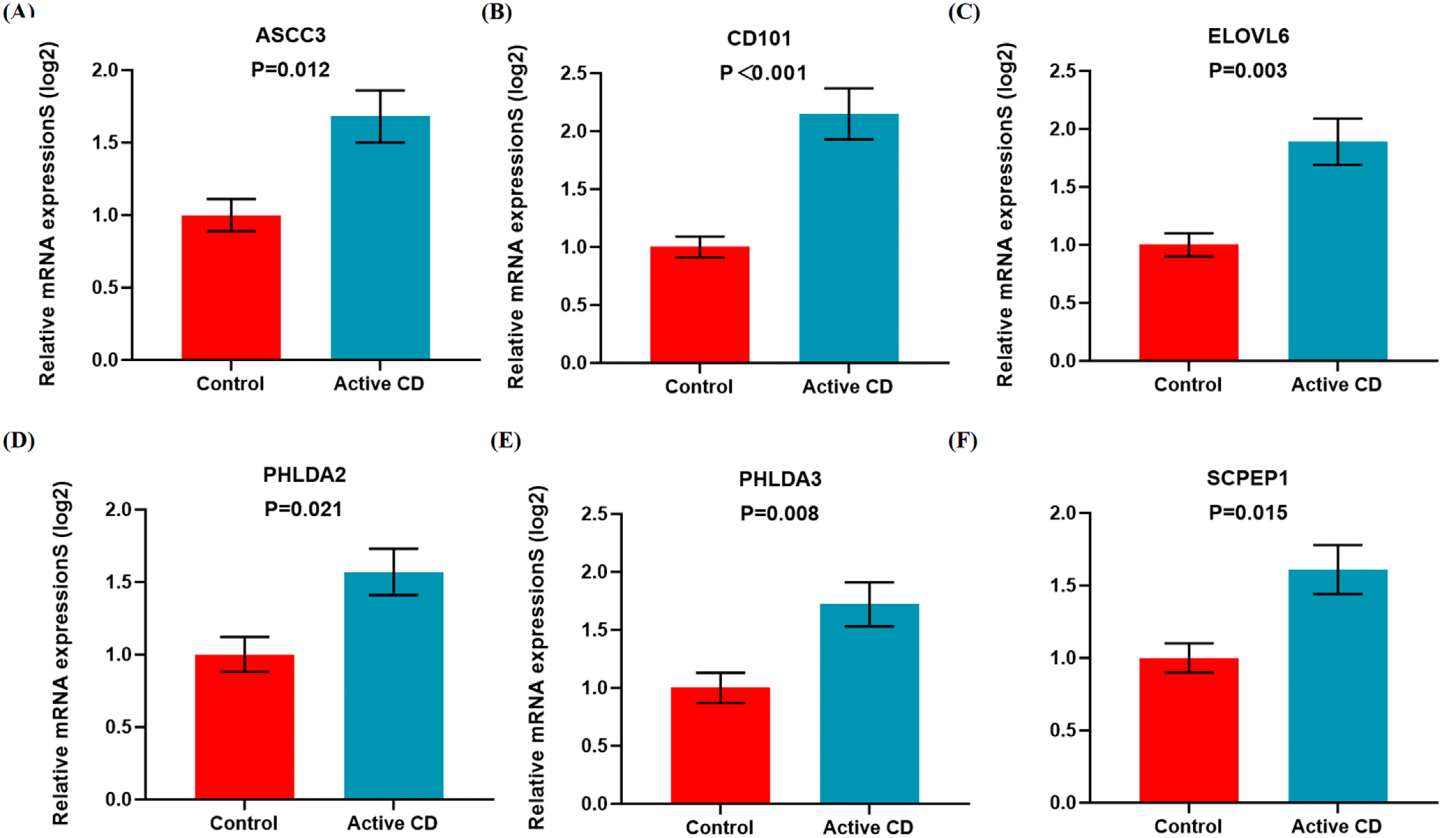
Validation of ASCC3, CD101, ELOVL6, PHLDA2, PHLDA3, and SCPEP1 expression in inactive CD by qPCR. (A) ASCC3 mRNA expression; (B) CD101 mRNA expression; (C) ELOVL6 mRNA expression; (D) PHLDA2 mRNA expression; (E) PHLDA3 mRNA expression; (F) SCPEP1 mRNA expression.

Examining the top 100 DEGs in the CD RNA‐seq dataset GSE217163 for active samples, we found that 19 genes show differential expression levels in CD. The differential expression levels of these genes reveal significant changes in their expression profiles, with *p*‐values ranging from 0.00038 for CD101 to 0.0376 for HMGCR. Specifically, CD101 and ELOVL6 show the lowest *p*‐values, indicating that they are the most significantly differentially expressed genes in CD, followed by PHLDA3, ASCC3, and several other genes including PHLDA2, SCPEP1, PNPLA3, PRELP, SQLE, SC5D, DCT, FASN, TNFRSF10D, IDI1, HMGCS1, S100A16, PLK2, SPATA18, and HMGCR, with their expression levels altered in the context of CD (Figure 8A–F). qPCR validation further confirmed the significant upregulation of ASCC3, CD101, ELOVL6, PHLDA2, PHLDA3, and SCPEP1 in intestinal mucosal samples from 24 patients with active CD compared to 20 healthy controls (all *p* < 0.05), consistent with the RNA‐seq findings (Figure 9A–E).

### 3.6 Causal Relationships Identified in CD Using TwoSampleMR and MRMix

**FIGURE 10:**
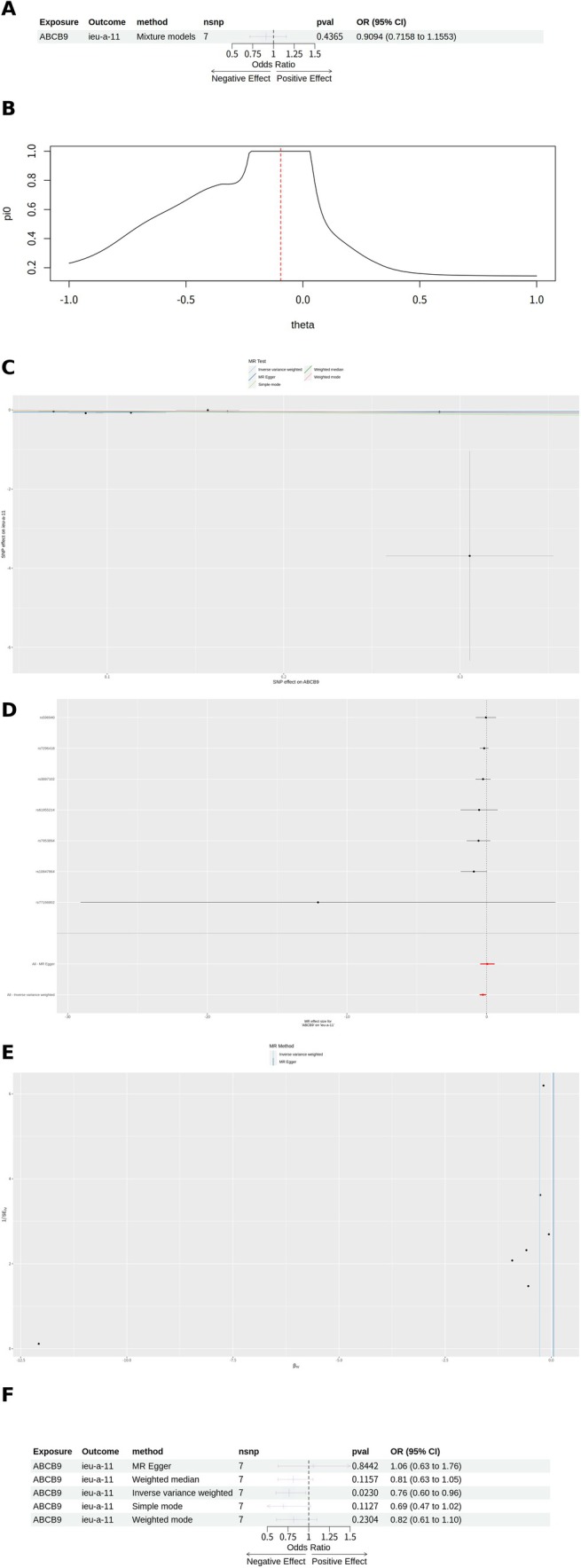
Causal Relationships Identified between ABCB9 and CD in dataset ieu‐a‐10. (A) Forest plot generated by MRMix. (B) Pi0‐theta plot generated by MRMix. (C) Scatter plot with TwoSampleMR. (D) Forest plot with TwoSampleMR. (E) Funnel plot with TwoSampleMR. (F) Forest plot generated by forestploter.

**FIGURE 11:**
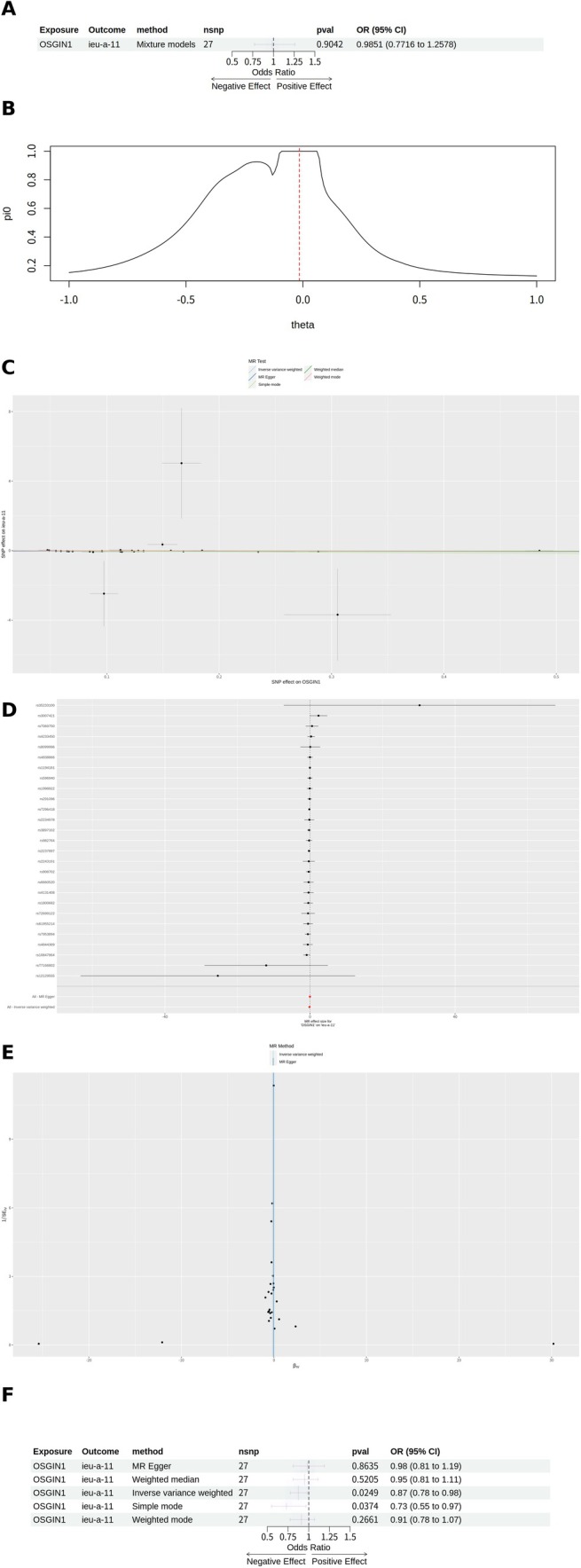
Causal Relationships Identified between OSGIN1 and CD on dataset ieu‐a‐10. (A) Forest plot generated by MRMix. (B) Pi0‐theta plot generated by MRMix. (C) Scatter plot with TwoSampleMR. (D) Forest plot with TwoSampleMR. (E) Funnel plot with TwoSampleMR. (F) Forest plot generated by forestploter.

The analysis of instrumental variable genes in relation to CD using TwoSampleMR and MRMix yielded distinct results. In the dataset ieu‐a‐10, TwoSampleMR identified two genes, ABCB9 (Figure 10C–F) and OSGIN1 (Figure 11C–F), as having a significant causal relationship with CD. However, when employing MRMix, only OSGIN1 (Figure 11A,B) showed a significant causal connection. The Mendelian randomization calculations revealed varying degrees of association, with TwoSampleMR reporting an inverse variance weighted estimate of −0.27 for ABCB9 and −0.13 for OSGIN1, suggesting a potential causal relationship with CD. In contrast, MRMix yielded a mixture model estimate of −0.24 for OSGIN1, indicating a weaker association. These findings highlight how different analytical approaches can influence the identification of causal relationships between genetic variants and complex diseases like CD.

**FIGURE 12:**
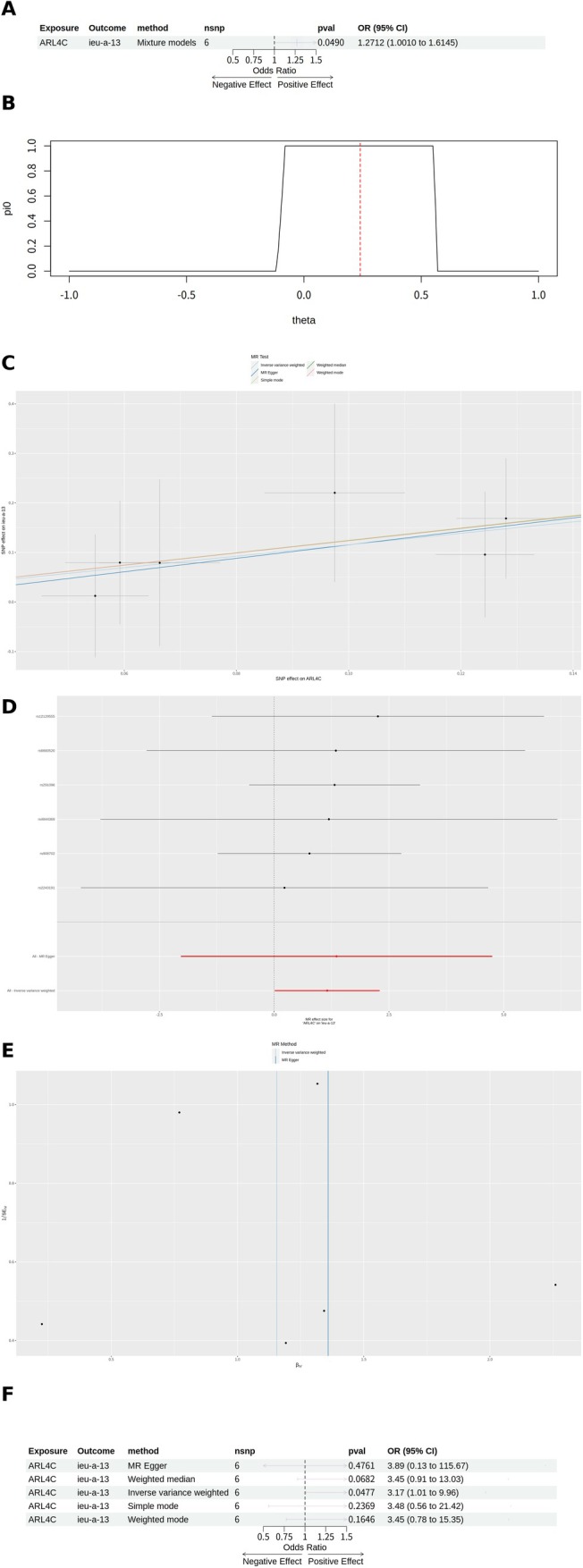
Causal Relationships Identified between ARL4C and CD on dataset ieu‐a‐11. (A) Forest plot generated by MRMix. (B) Pi0‐theta plot generated by MRMix. (C) Scatter plot with TwoSampleMR. (D) Forest plot with TwoSampleMR. (E) Funnel plot with TwoSampleMR. (F) Forest plot generated by forestploter.

**FIGURE 13:**
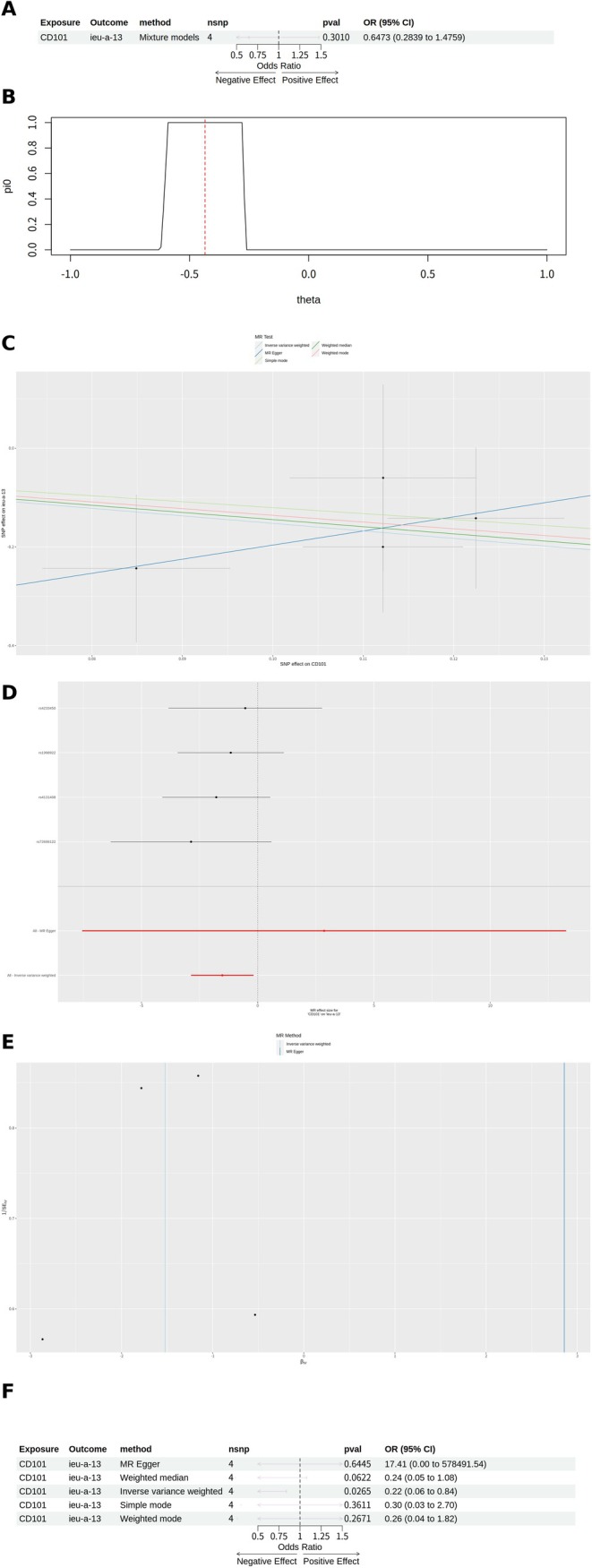
Causal Relationships Identified between CD101 and CD on dataset ieu‐a‐11. (A) Forest plot generated by MRMix. (B) Pi0‐theta plot generated by MRMix. (C) Scatter plot with TwoSampleMR. (D) Forest plot with TwoSampleMR. (E) Funnel plot with TwoSampleMR. (F) Forest plot generated by forestploter.

**FIGURE 14:**
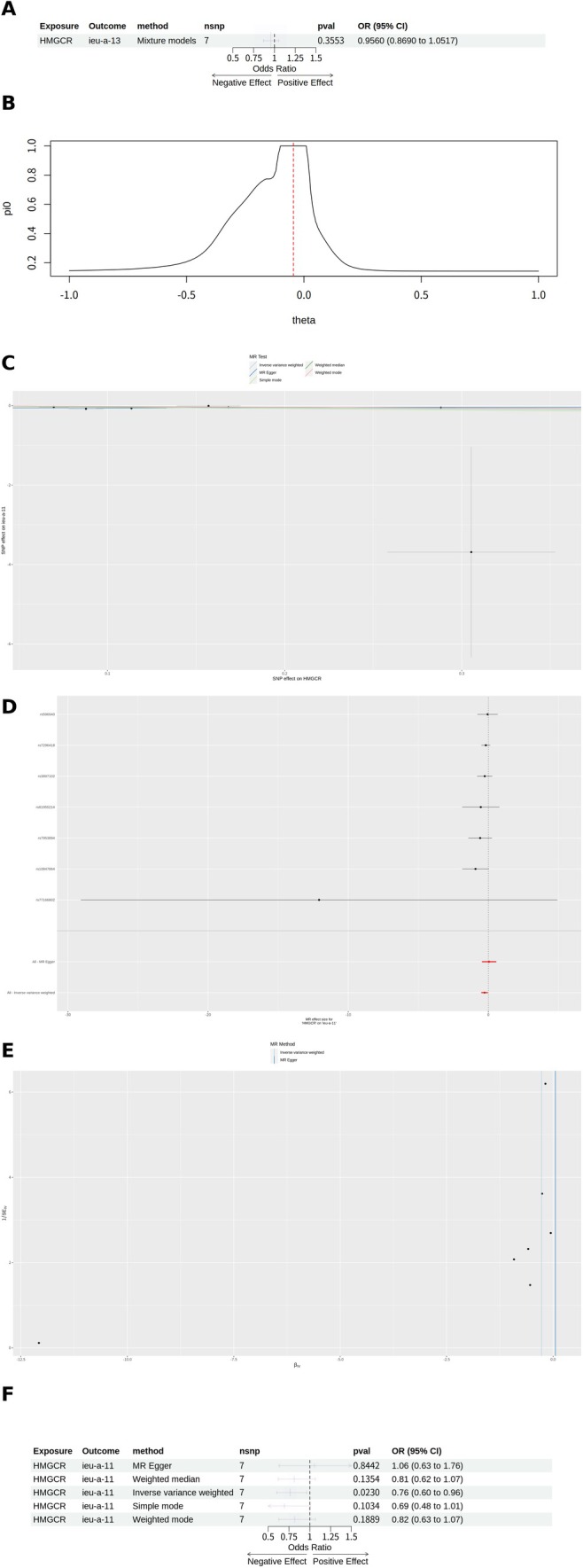
Causal Relationships Identified between HMGCR and CD on dataset ieu‐a‐11. (A) Forest plot generated by MRMix. (B) Pi0‐theta plot generated by MRMix. (C) Scatter plot with TwoSampleMR. (D) Forest plot with TwoSampleMR. (E) Funnel plot with TwoSampleMR. (F) Forest plot generated by forestploter.

**FIGURE 15:**
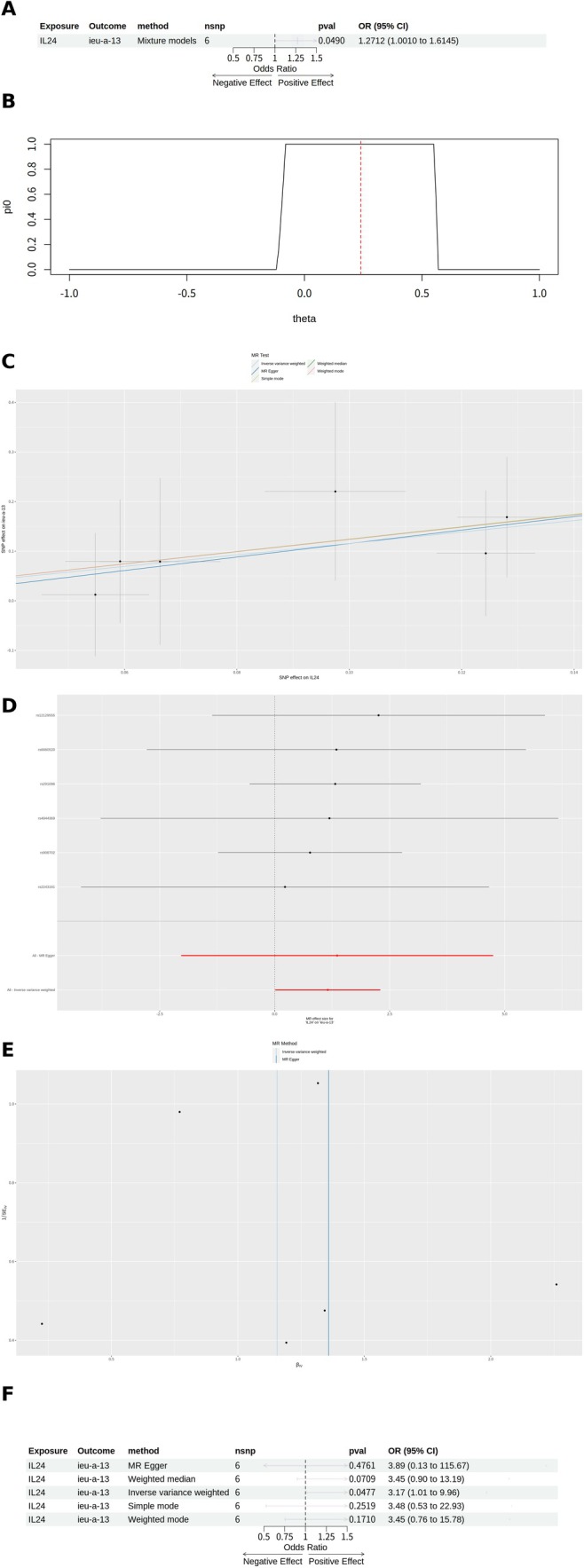
Causal Relationships Identified between IL24 and CD on dataset ieu‐a‐11. (A) Forest plot generated by MRMix. (B) Pi0‐theta plot generated by MRMix. (C) Scatter plot with TwoSampleMR. (D) Forest plot with TwoSampleMR. (E) Funnel plot with TwoSampleMR. (F) Forest plot generated by forestploter.

In the dataset ieu‐a‐11, TwoSampleMR identified four genes, ARL4C (Figure 12C–F), CD101 (Figure 13C–F), HMGCR (Figure 14A–F), and IL24 (Figure 15C–F), as having a significant causal relationship with CD. On the other hand, MRMix identified three genes, ARL4C (Figure 12A,B), CD101 (Figure 13A,B), and IL24 (Figure 15A,B), as significantly associated with CD. The estimated odds ratios from both methods ranged from 1.27 to 2.48, indicating a moderate to strong association between these instrumental variables and CD. These results provide valuable insights into the genetic determinants of CD and underscore the importance of further research.

**FIGURE 16:**
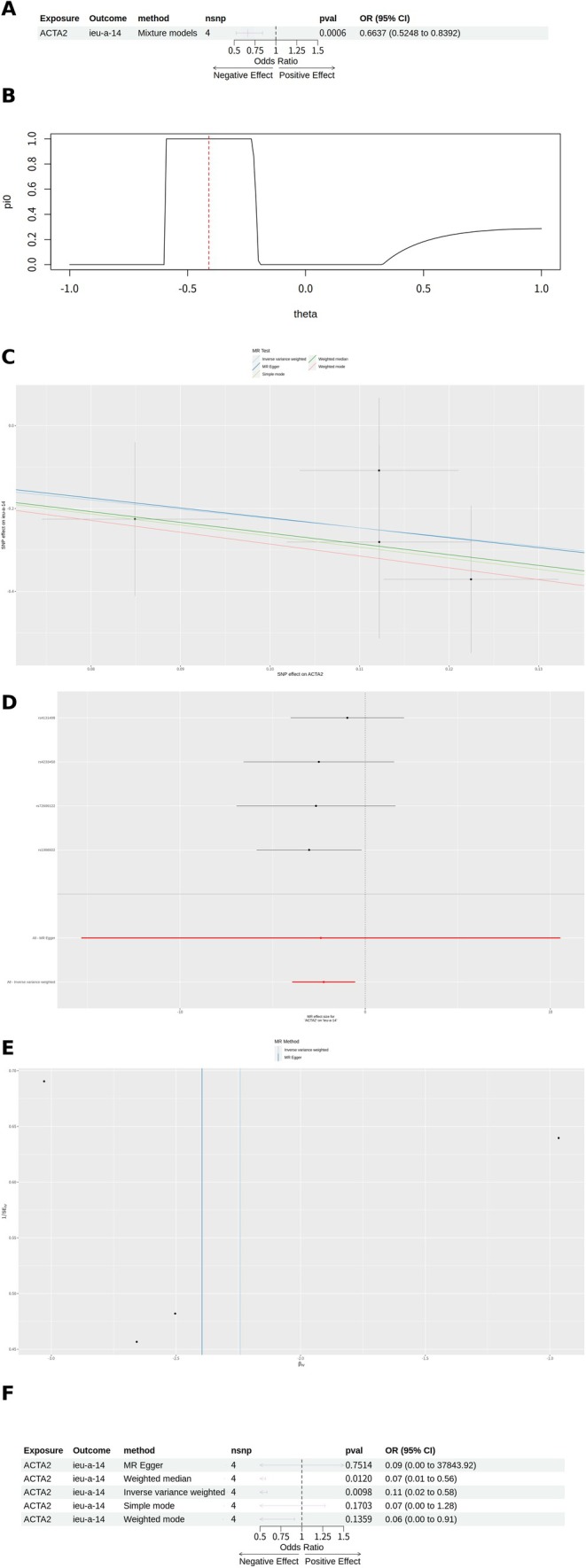
Causal Relationships Identified between ACTA2 and CD on dataset ieu‐a‐13. (A) Forest plot generated by MRMix. (B) Pi0‐theta plot generated by MRMix. (C) Scatter plot with TwoSampleMR. (D) Forest plot with TwoSampleMR. (E) Funnel plot with TwoSampleMR. (F) Forest plot generated by forestploter.

**FIGURE 17:**
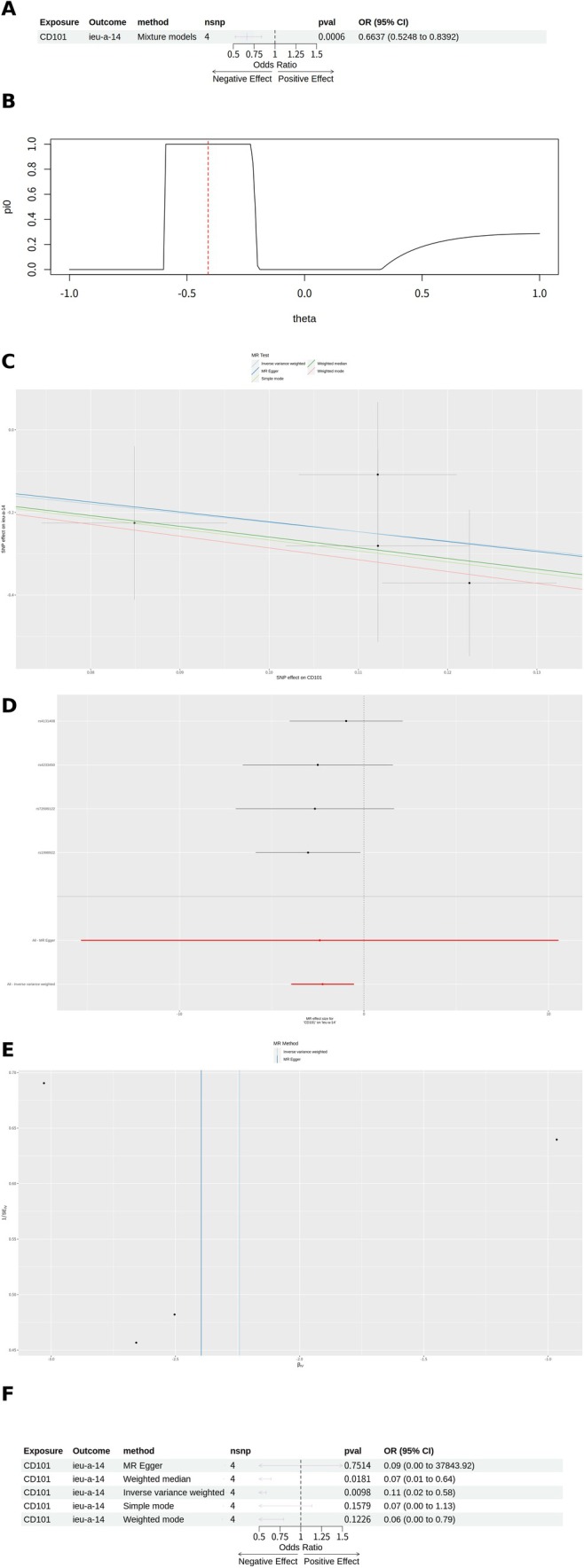
Causal Relationships Identified between CD101 and CD on dataset ieu‐a‐13. (A) Forest plot generated by MRMix. (B) Pi0‐theta plot generated by MRMix. (C) Scatter plot with TwoSampleMR. (D) Forest plot with TwoSampleMR. (E) Funnel plot with TwoSampleMR. (F) Forest plot generated by forestploter.

**FIGURE 18:**
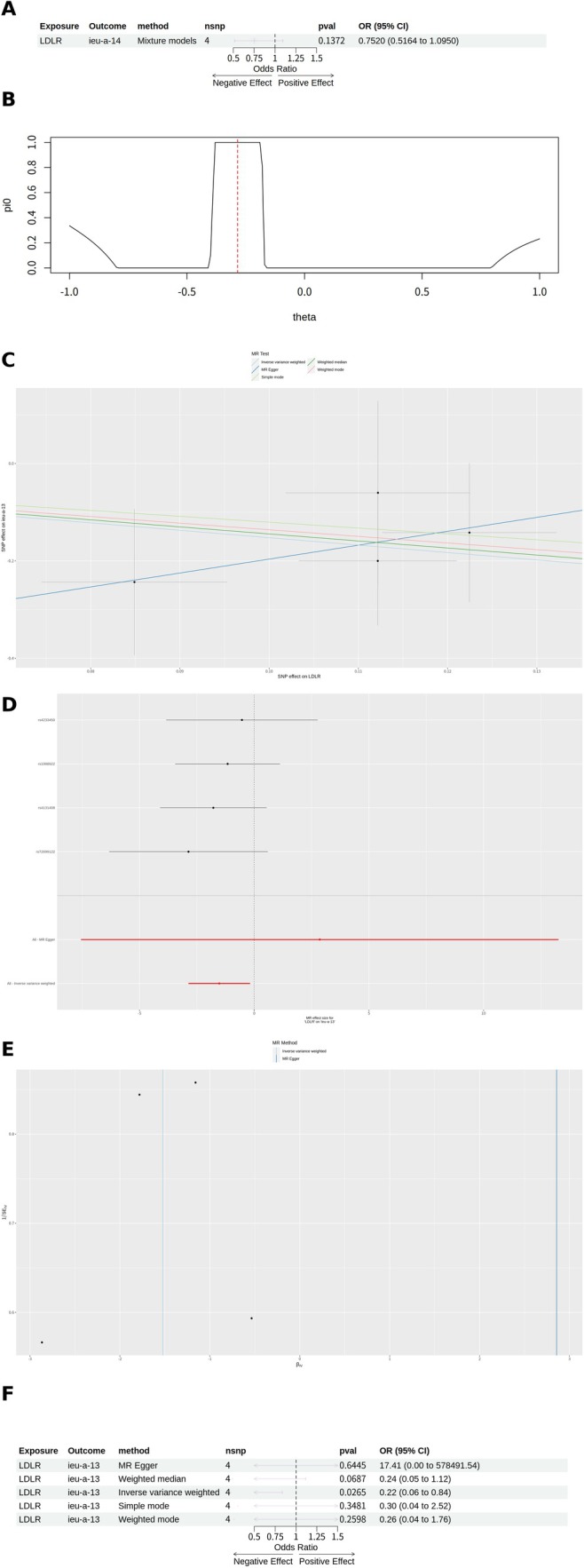
Causal Relationships Identified between LDLR and CD on dataset ieu‐a‐13. (A) Forest plot generated by MRMix. (B) Pi0‐theta plot generated by MRMix. (C) Scatter plot with TwoSampleMR. (D) Forest plot with TwoSampleMR. (E) Funnel plot with TwoSampleMR. (F) Forest plot generated by forestploter.

In the analysis of dataset ieu‐a‐13, TwoSampleMR identified three genes, ACTA2 (Figure 14C–F), CD101 (Figure 17C–F), and LDLR (Figure 18A–F), as having a significant causal relationship with CD. ACTA2 and CD101 were found to have an estimated causal effect of −0.41, corresponding to a 0.66‐fold decrease in the odds of developing CD. However, LDLR did not show a significant causal relationship using TwoSampleMR. In contrast, MRMix identified only ACTA2 (Figure 16A,B) and CD101 (Figure 17A,B) as significant, with both genes exhibiting an estimated causal effect of −0.41. These findings suggest that ACTA2 and CD101 are instrumental variables for establishing causality between genetic variants and CD.

## 4 Discussion

The study's findings provide significant insights into the molecular responses induced by H_2_O_2_ intervention, specifically through the analysis of DEGs. The identification of the top 10 up‐regulated and down‐regulated DEGs highlights potential pathways that may be involved in oxidative stress and its related pathologies, particularly within the gastrointestinal system and more specifically in diseases such as CD. Among the up‐regulated DEGs, GDF15 (Growth Differentiation Factor 15) exhibited the highest log fold change. This gene is known for its role in regulating inflammation and apoptosis under stress conditions, including oxidative stress induced by H_2_O_2_. Elevated GDF15 levels have been associated with chronic inflammatory conditions, including CD. A recent study demonstrated that GDF15 levels are significantly higher in patients with CD compared to non‐inflammatory controls, suggesting its role as a potential biomarker for disease severity and progression [18]. The connection between GDF15 and CD underlines its importance in maintaining intestinal homeostasis and the potential impact of oxidative stress on exacerbating inflammatory responses in the gut. CDKN1A (p21) was another prominently up‐regulated gene in this study, known for its role in cell cycle regulation and response to DNA damage. Its upregulation under oxidative stress conditions, such as those induced by H_2_O_2_, aligns with its protective role in inhibiting cell cycle progression in response to DNA damage. This gene has also been implicated in the pathogenesis of CD, where dysregulated cell cycle control contributes to the chronicity of inflammation and fibrosis [19]. MDM2 is another key gene identified in this study, which is involved in the negative regulation of p53, a tumour suppressor protein that plays a critical role in apoptosis and genomic stability. Elevated levels of MDM2 have been associated with various cancers and inflammatory conditions. In the context of CD, aberrant p53 signalling has been linked to defective apoptosis in intestinal epithelial cells, contributing to the disease's pathogenesis [20]. The upregulation of MDM2 under oxidative stress conditions could imply a protective response aimed at controlling excessive apoptosis, yet it may also contribute to the persistence of inflammation in chronic conditions like CD.

On the other hand, among the down‐regulated DEGs, DCT (dopachrome tautomerase) and HMGCS1 (3‐hydroxy‐3‐methylglutaryl‐CoA synthase 1) showed significant suppression. DCT is involved in melanin synthesis and has been recently implicated in immune regulation. The suppression of DCT might suggest a downregulation of melanogenesis‐related pathways, potentially impacting immune responses in the gut, which could be relevant in the context of CD, where immune dysregulation is a key feature [21]. HMGCS1 plays a central role in cholesterol biosynthesis. The downregulation of this gene under oxidative stress might indicate a shift in metabolic processes, which is relevant in the context of CD, where altered lipid metabolism has been observed [22]. CDH1 (E‐cadherin) was also among the top down‐regulated DEGs, which is noteworthy given its crucial role in maintaining epithelial integrity. Downregulation of CDH1 has been linked to epithelial‐mesenchymal transition (EMT), a process implicated in fibrosis and stricture formation in CD [23].

The current study delves into the intricate relationship between oxidative stress, particularly mediated by H_2_O_2_, and the activation of specific GO pathways in the context of digestive system diseases, with a particular focus on CD. Through a comprehensive GO and KEGG pathway analysis, a total of 109 GO‐BP pathways were identified, highlighting critical roles in sterol biosynthesis, metabolism, and organic hydroxy compound biosynthesis. Notably, these pathways were significantly enriched, with p.adjust values below 0.05, underscoring their potential involvement in the pathophysiology of CD. Oxidative stress, mediated by reactive oxygen species (ROS) like H_2_O_2_, has been increasingly implicated in the pathogenesis of inflammatory bowel diseases (IBD), including CD. The enrichment of pathways such as the sterol biosynthetic process (GO:0016126) and sterol metabolic process (GO:0016125) in our study suggests a pivotal role for sterol metabolism in the response to oxidative stress. Previous research has shown that oxidative stress can disrupt cholesterol homeostasis, which is tightly regulated under normal physiological conditions but becomes dysregulated during chronic inflammation, as observed in IBD [24]. The identification of four cellular component pathways, including the cyclin‐dependent protein kinase holoenzyme complex (GO:0000307) and the serine/threonine protein kinase complex (GO:1902554), further implicates kinase signalling in the cellular response to oxidative stress. These kinase complexes play critical roles in cell cycle regulation and apoptosis, processes that are often dysregulated in chronic inflammatory conditions like CD. The activation of these complexes in response to oxidative stress suggests that they may serve as mediators of the cellular response to oxidative damage, potentially exacerbating the inflammatory milieu observed in CD [25].

In the GO‐MF analysis, the enrichment of pathways related to oxidoreductase activity (GO:0016705) and iron ion binding (GO:0005506) indicates a direct involvement of redox reactions in the cellular response to oxidative stress. Oxidoreductases, which catalyse electron transfer reactions, are crucial in mitigating oxidative damage by neutralizing ROS. However, when overwhelmed by excessive ROS production, as can occur during chronic inflammation, these enzymes may contribute to oxidative damage to lipids, proteins, and DNA, further perpetuating the inflammatory cycle seen in CD [26]. Our findings resonate with previous studies that have linked oxidative stress and mitochondrial dysfunction with the pathogenesis of CD [27]. The enrichment of pathways involved in sterol metabolism and kinase signalling further supports the hypothesis that oxidative stress not only contributes to the initial onset of CD but also plays a role in its chronicity. The dysregulation of sterol biosynthesis and the activation of protein kinase complexes suggest potential therapeutic targets for modulating oxidative stress in CD. Indeed, targeting these pathways may help to restore redox balance and mitigate the inflammatory response, offering new avenues for treatment.

Building on these findings, our GSVA analysis further illuminated the impact of oxidative stress on various GO pathways. The analysis revealed significant results across 11 GO pathways, such as “Regulation of DNA‐Directed DNA Polymerase Activity,” “Proteasome Regulatory Particle,” and “Proteasome Core Complex.” These pathways were found to have positive GSVA scores, suggesting their upregulation in response to oxidative stress. The regulation of DNA‐directed DNA polymerase activity is crucial in maintaining genomic stability during oxidative stress, which could be particularly relevant in the context of CD, where inflammation‐driven DNA damage is a common feature. The upregulation of proteasome‐related pathways further underscores the role of protein degradation and turnover in managing oxidative damage, a process that is vital for cellular survival in an inflamed and oxidative environment such as that observed in CD [28, 29]. Additionally, our study identified pathways such as “Myeloid Dendritic Cell Differentiation,” which had a moderate GSVA score but a highly significant associated value. This pathway is particularly relevant given the known role of dendritic cells in the immune dysregulation observed in CD. Oxidative stress may exacerbate this dysregulation, promoting an environment conducive to chronic inflammation. The pathway “Cellular Response to Increased Oxygen Levels” also demonstrated a significant association with oxidative stress, despite having a lower GSVA score. This finding is consistent with previous research indicating that oxidative stress can lead to the activation of oxygen‐sensing pathways, which in turn modulate inflammatory responses in diseases like CD [30]. The significance of this pathway suggests a potential mechanism by which oxidative stress exacerbates the inflammatory milieu in CD, potentially contributing to disease progression and severity. Furthermore, pathways related to “Platelet Dense Granule” and “Proton Transporting Two‐Sector ATPase Complex Catalytic Domain” showed negative GSVA scores, indicating possible downregulation. This downregulation could reflect a compensatory response to the upregulation of oxidative stress‐related pathways, as the body attempts to mitigate the damaging effects of prolonged oxidative stress. The involvement of these pathways in energy metabolism and cellular transport processes further highlights the intricate balance between oxidative stress and cellular homeostasis, which may be disrupted in CD [31].

In this study, we also identified 22 KEGG pathways with a threshold of p.adjust < 0.05 using DESeq2 and clusterProfiler, with significant associations observed in pathways such as the p53 signalling pathway, steroid biosynthesis, and the FoxO signalling pathway. The top five KEGG pathways (hsa04115, hsa00100, hsa04068, hsa05216, hsa05218) were further visualized using ggkegg, highlighting their relevance in our analysis. Oxidative stress has been shown to play a significant role in the pathogenesis of CD, contributing to the chronic inflammation characteristic of this condition. The FoxO signalling pathway (hsa04068), identified in our KEGG pathway analysis, is known to be crucial in regulating oxidative stress responses. The FoxO transcription factors are involved in the regulation of genes responsible for cellular defence mechanisms against oxidative damage, including those encoding antioxidant enzymes such as catalase and superoxide dismutase (SOD) [32]. The upregulation of these genes can mitigate the detrimental effects of reactive oxygen species (ROS) like H_2_O_2_, which is particularly relevant in the context of inflammatory bowel diseases (IBD), including CD. Moreover, the p53 signalling pathway (hsa04115), another pathway highlighted in our findings, is well‐known for its role in DNA repair and apoptosis in response to cellular stress. p53 can induce the expression of genes involved in antioxidant defence, thus providing a protective mechanism against oxidative stress‐induced damage [33]. Steroid biosynthesis (hsa00100) also emerged as a significant pathway in our analysis. Steroids are known to have anti‐inflammatory effects, and their biosynthesis is crucial in modulating the inflammatory response in diseases like CD. The disruption of steroid biosynthesis could exacerbate the inflammatory processes, contributing to the severity of the disease [34]. In the context of CD, the chronic inflammation and oxidative stress observed could be linked to these KEGG pathways. For instance, previous studies have shown that patients with CD exhibit altered lipid metabolism, which is intricately connected with steroid biosynthesis pathways. These disruptions can lead to an imbalance in the production of anti‐inflammatory steroids, further aggravating the inflammatory state [35]. Furthermore, the FoxO signalling pathway's involvement in lipid metabolism and energy homeostasis also points to its potential role in the metabolic alterations observed in CD. Dysregulated lipid metabolism can contribute to the chronic inflammation seen in CD, as lipids play a crucial role in modulating immune responses [36]. Overall, our findings provide insights into the complex interplay between oxidative stress, specifically H_2_O_2_‐mediated signalling, and gastrointestinal diseases like CD. The involvement of key KEGG pathways, including the p53 signalling pathway, steroid biosynthesis, and the FoxO signalling pathway, underscores the potential mechanisms through which oxidative stress may contribute to the pathogenesis and progression of CD. These pathways could serve as potential therapeutic targets for mitigating oxidative stress and managing CD more effectively. Further research is warranted to explore the precise molecular mechanisms linking these pathways to the clinical manifestations of CD and to develop targeted interventions that could improve patient outcomes.

In this study, we investigated the differential expression of genes in CD patients, focusing on the effects of H_2_O_2_ intervention, which is known to induce oxidative stress, a key factor in the pathogenesis of various diseases, including CD. Our analysis revealed several DEGs in both active CD and inactive CD states of CD, suggesting that oxidative stress might play a crucial role in modulating gene expression related to disease pathogenesis. Notably, we further validated the mRNA expression of key genes using qPCR: FASN and HMGCR were significantly upregulated in inactive CD, while ASCC3, CD101, ELOVL6, PHLDA2, PHLDA3, and SCPEP1 were markedly increased in active CD (all *p* < 0.05), confirming the RNA‐seq findings. One of the most significant findings was the downregulation of FASN (Fatty Acid Synthase) in inactive CD patients, with a *p*‐value of 0.0032. FASN is critical in lipid biosynthesis, and its downregulation has been associated with impaired lipid metabolism, which could exacerbate the inflammatory processes in CD. The significant upregulation of FASN at both transcriptomic and qPCR levels further supports its potential role in persistent oxidative stress and metabolic dysregulation during disease remission. The involvement of FASN in CD has been supported by other studies as well. For instance, the study by Wu et al., 2024 demonstrated that FASN downregulation due to AhR (Aryl Hydrocarbon Receptor) activation led to reduced fatty acid synthesis and alleviated intestinal fibrosis, a common complication in CD [37]. This suggests that oxidative stress‐mediated downregulation of FASN might be part of a protective response, potentially mitigating excessive lipid accumulation and fibrosis in CD. Another gene of interest is CD101, which exhibited significant differential expression in active CD patients, with a *p*‐value of 0.00038. CD101 is known to inhibit the expansion of colitogenic T cells and promote regulatory T cell function, thereby suppressing inflammatory responses in the gut [38]. Reduced expression of CD101 has been correlated with enhanced IL‐17 production and increased disease activity in CD patients. This gene's differential expression under oxidative stress conditions further underscores its role in maintaining immune homeostasis in CD. Additionally, ELOVL6, involved in fatty acid elongation, was significantly differentially expressed in active CD patients, suggesting its role in lipid metabolism and inflammation. Other notable DEGs include HMGCR (3‐Hydroxy‐3‐Methylglutaryl‐CoA Reductase) and PRELP (Proline/Arginine Rich End Leucine Rich Repeat Protein). HMGCR is a key enzyme in cholesterol biosynthesis and has been implicated in the pathogenesis of CD through its role in lipid metabolism. Genetic studies have shown that inhibition of HMGCR, mimicking the effects of statins, does not significantly affect immune‐related diseases like CD, although its role in lipid regulation remains crucial [39]. PRELP, known for its role in maintaining extracellular matrix (ECM) integrity, also showed differential expression in both inactive CD and active CD patients. The downregulation of PRELP could contribute to ECM remodelling and fibrosis, common features of chronic inflammation in CD [40]. Emerging evidence has linked sorting nexin 10 (SNX10) to intestinal inflammation and oxidative stress, which is highly relevant to our findings. A recent study demonstrated that SNX10 expression in human intestinal tissues is significantly upregulated and positively correlated with the severity of Crohn's disease [41]. Consistently, increased SNX10 levels were also observed in mouse colitis models, accompanied by enhanced oxidative stress and inflammatory responses. Mechanistically, SNX10 promotes reactive oxygen species (ROS) accumulation, activates the NF‐κB signalling pathway, and impairs intestinal epithelial barrier function, thereby exacerbating mucosal inflammation. Given the central role of oxidative stress in CD pathogenesis, SNX10 may serve as a critical regulator linking oxidative stress, genetic susceptibility, and disease severity. Although SNX10 was not identified in our current multi‐omics analysis, its established role in modulating oxidative stress and intestinal inflammation suggests that integrating SNX10‐related pathways into future studies will further deepen our understanding of CD pathogenesis.

Overall, the differential expression of H_2_O_2_ intervention‐related DEGs in CD patients underscores the complex interplay between oxidative stress and digestive system diseases, particularly CD. The involvement of key genes such as FASN, CD101, ELOVL6, and HMGCR in oxidative stress responses and lipid metabolism highlights potential therapeutic targets for managing CD. Further research is needed to explore these pathways in greater detail, with the aim of developing targeted interventions that could mitigate oxidative stress and improve patient outcomes in CD.

## 5 Limitations

Despite the significant findings of this study, there are several limitations that need to be acknowledged. First, the study primarily relied on RNA‐seq data and GWAS datasets, which, although powerful, may not capture the entire complexity of gene–environment interactions in CD. The study's focus on oxidative stress‐related DEGs provides a narrow view, potentially overlooking other crucial pathways involved in CD pathogenesis. Additionally, the use of MR analysis assumes that the selected genetic variants are valid instrumental variables, free from pleiotropy or confounding, which might not always hold true, thus introducing potential biases. Furthermore, the datasets used in this study are based on specific populations, which may limit the generalizability of the findings to other ethnic groups. Lastly, while the study identified potential therapeutic targets, functional validation of these targets in experimental or clinical settings was not conducted, leaving a gap between the identified genetic associations and their actual biological significance in CD. Third, the current study only validated the mRNA expression of key genes using qPCR, while corresponding protein‐level verification by Western blot (WB) was not performed. Although qPCR confirmed the transcriptional changes consistent with RNA‐seq data, protein expression may not fully mirror mRNA levels due to post‐transcriptional and post‐translational regulation. Therefore, further WB validation of these proteins in larger clinical cohorts will be necessary in future studies to strengthen the findings.

## 6 Conclusion

This study provides important insights into the molecular networks underlying oxidative stress in Crohn's disease, highlighting the significant role of specific DEGs such as FASN, CD101, and HMGCR in disease pathogenesis. Through the integration of multi‐omics data and Mendelian Randomization analysis, the study underscores the potential of targeting oxidative stress‐related pathways for therapeutic interventions in CD. qPCR validation confirmed that FASN and HMGCR were persistently upregulated in inactive CD, while ASCC3, CD101, ELOVL6, PHLDA2, PHLDA3, and SCPEP1 were significantly elevated in active CD, reinforcing the link between oxidative stress and disease activity. These findings pave the way for future research aimed at validating these molecular targets and developing effective strategies to manage oxidative stress in CD patients.

## Author Contributions

**Yuxiu Yang:** writing – original draft, writing – review and editing. **Juan Yang:** writing – review and editing, writing – original draft, data curation. **Lida Zhang:** writing – review and editing, methodology. **Xiaqing Wang:** writing – original draft, methodology.

## Funding

The authors have nothing to report.

## Ethics Statement

This study was conducted in accordance with the Declaration of Helsinki and approved by the Ethics Committee of FuWai Central China Cardiovascular Hospital.

## Consent

All authors have agreed to publish.

## Conflicts of Interest

The authors declare no conflicts of interest.

## Data Availability

The datasets used and/or analyzed during the current study are available from the corresponding author on reasonable request.
